# Tolosa-Hunt Syndrome: A Case Report

**DOI:** 10.31729/jnma.5700

**Published:** 2021-06-30

**Authors:** K.C. Siddhanta, K.C. Shreeyanta, Prajjwal Kunwar, Krishna Dhungana

**Affiliations:** 1 Kathmandu Medical College and Teaching Hospital, Sinamangal, Kathmandu, Nepal; 2 Dirghayu Guru Hospital, Chabahil, Kathmandu, Nepal; 3 HAMS Hospital, Dhumbarahi, Kathmandu, Nepal; 4 Department of Neurology, Kathmandu Medical College and Teaching Hospital, Sinamangal, Kathmandu, Nepal

**Keywords:** *painful ophthalmoplegia*, *ptosis*, *Tolosa-Hunt Syndrome*

## Abstract

Tolosa-Hunt Syndrome is a rare disease characterized by painful ophthalmoplegia affecting third, fourth, and/or sixth cranial nerve caused by non-specific inflammation in the cavernous sinus or superior orbital fissure of unknown etiology. We presented a 67-year-old female with Tolosa-Hunt Syndrome. She had a right-sided headache and periorbital pain with double vision. Examination showed right-sided ptosis, right-sided trochlear and abducens nerve palsy, and partial right-sided oculomotor nerve palsy with hypoesthesia in the area of the ophthalmic division of the trigeminal nerve. Magnetic resonance imaging of head and orbit showed altered signal intensity changes in the optic nerve and lateral rectus muscle. After steroid therapy, pain and ptosis were significantly improved in 72 hours. Tolosa-Hunt Syndrome is a diagnosis of exclusion, with clinical presentation, normal investigations, magnetic resonance imaging findings, and response to steroid therapy crucial in making the diagnosis.

## INTRODUCTION

Tolosa-Hunt syndrome (THS) is a painful ophthalmoplegia, usually unilateral, caused by nonspecific inflammation of the cavernous sinus or superior orbital fissure.^1^ It is one of the rare disorders recognized by the National Organization for Rare Disorders (NORD) and is also included as one of the painful cranial neuropathies by the International Headache Society (IHS) in its headache classification.^2^ The estimated annual incidence is one case per million per year. It presents with unilateral orbital/periorbital pain associated with paralysis of one or more of the third, fourth, or sixth cranial nerve.^3^ THS is a diagnosis of exclusion and it responds well to steroids.^4^

## CASE REPORT

A 67-year-old female presented to Neurology outpatient department with complaints of right-sided headache and double vision for 3 weeks. Headache was sudden in onset, pricking around the right eye and throbbing in the right frontal region, continuous, associated with blurry vision and numbness over the right frontal region. Headache was followed by double vision 1 day later, which was progressive, associated with drooping of the right eyelid for 1 week. The patient was under medication for hypertension. She was nondiabetic, non-smoker, with no history of head trauma.

**Figure 1:**
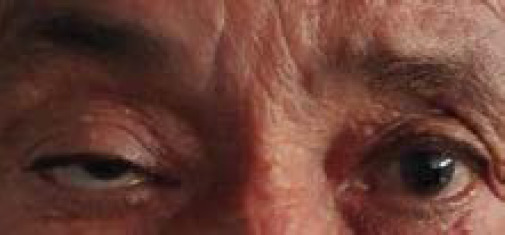
Right upper lid ptosis.

**Figure 2:**
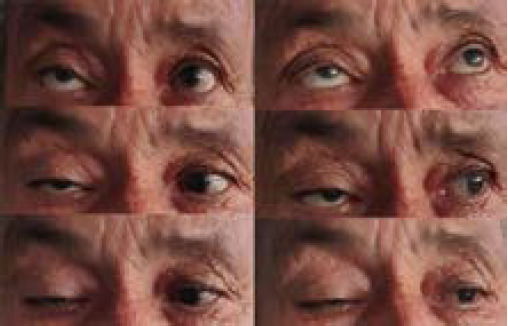
Neuro-ophthalmologic examination on day 1 of treatment shows right upper lid ptosis, paresis of the third, fourth, and sixth right cranial nerves.

On examination, the patient was conscious, cooperative, and orientated. Vitals were within normal range. Ocular examination of the right eye revealed right upper lid ptosis, complete weakness of depression and abduction while adduction and elevation were limited to 50% normal, visual acuity 6/9, relative apparent pupillary defect present, pupil ~2mm and sluggishly reactive to light, other findings were normal (Figure 1 and 2).

Examination of the left eye showed visual acuity 6/12, other findings were unremarkable. Neurological examination revealed right oculomotor, trochlear, and abducens nerve paresis, with hypoesthesia over area supplied by right ophthalmic division of trigeminal nerve. All other physical and systemic examinations were normal.

On investigation, hematology report showed mild thrombocytosis (443000/mm^3^), hemoglobin of 13.4mg%, erythrocyte sedimentation rate 14mm in the first hour, C-reactive protein 3.62mg/L. The biochemical report showed mild elevated total cholesterol of 207mg/dl and low-density lipoprotein of 124mg/dl. Her blood glucose, HbA_1c_, triglyceride, high-density lipoprotein, very low-density lipoprotein, urea, creatinine, sodium, potassium was normal. Lumbar puncture was done and the cerebrospinal fluid (CSF) study showed normal glucose, protein, lactate dehydrogenase, 3cells/mm^3^ (all lymphocytes). CSF culture and sensitivity, gram stain, potassium hydroxide mount, acid fast bacilli stain was negative. The anti-nuclear antibody test was negative. Serology including Human Immunodeficiency Virus, Hepatitis C Virus, Hepatitis B surface Antigen, and Venereal Disease Research Laboratory test (VDRL) was non-reactive.

**Figure 3:**
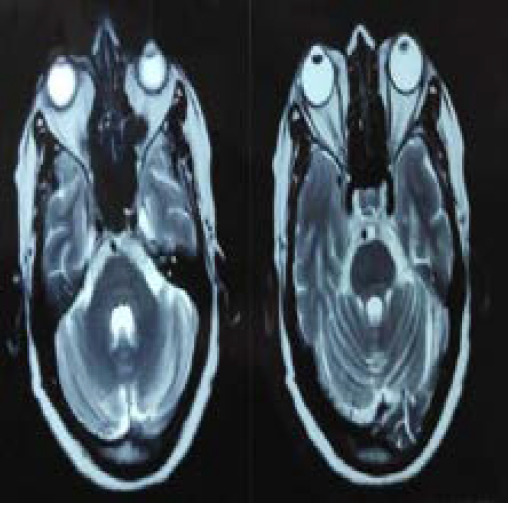
MRI of brain and orbit (Axial) showing increased right optic nerve diameter with altered signal intensity changes in optic nerve and lateral rectus muscle.

Magnetic Resonance Imaging (MRI) of the brain and orbit showed comparatively increased diameter (5.7mm) of the retrobulbar portion of the intra-orbital segment of the right optic nerve with T2 high signal intensity and altered signal intensity changes in the lateral aspect of the right globe in the region of the insertion site of lateral rectus muscle showing T2/STIR (short-tau inversion recovery) high signal intensity (Figure 3).

Mucosal thickening of the bilateral maxillary sinus and deviated nasal septum towards the left side was also noted. MRI of the brain with magnetic resonance angiography (MRA) and orbit done 10 days later following treatment showed improvement with normal MRA brain findings.

The patient was started on high dose intravenous methylprednisolone 1 gram/day for 3 days along with gastroprotective therapy. There was a significant reduction in right periorbital pain and ptosis after 72 hours with a slight improvement of ocular movement. The patient was discharged on day 3 with oral prednisolone 50mg daily for 11 days and follow-up in Neurology.

## DISCUSSION

Tolosa first described the condition in 1954, in a patient with unilateral recurrent painful ophthalmoplegia involving cranial nerves III, IV, VI, and V1.^1^ Similar cases were reported by Hunt et al. in 1961.^2^ Smith and Taxdal called it Tolosa-Hunt Syndrome for the first time in 1966.^2^ The latter authors stressed the importance of the dramatic rapid response to steroid therapy.^4^

Tolosa-Hunt syndrome is the nonspecific granulomatous inflammation characterized by infiltration of lymphocytes and plasma cells primarily in and around the cavernous sinus, with variable extension into and beyond the superior orbital fissure/ orbital apex.^1^ Etiology is still unknown however, traumatic injury, tumors, or an aneurysm could be the potential triggers.^2^ It does not have any age or sex predilection and can affect anyone in the first to the eighth decade of life.^5^ It is almost always unilateral (except in 4-5% of cases) and if bilateral, it shifts the balance in support of other differential diagnoses.^6^ It is considered a very benign illness, but exclusion of more malignant diseases bears utmost importance whenever any patient presents with such clinical features.^1^

**Table:** 

The International Classification of Headache Disorders (ICHD) criteria for THS include the following:^7^	
A.	Unilateral headache fulfilling criterion C
B.	Both of the following:
	1. granulomatous inflammation of the cavernous sinus, superior orbital fissure or orbit, demonstrated by MRI or biopsy
	2. paresis of one or more of the ipsilateral III^rd^, IV^th^, and/or VI^th^ cranial nerves
C.	Evidence of causation demonstrated by both of the following:
	1. headache has preceded paresis of the III^rd^, IV^th^, and/or VI^th^ nerves by ≤2 weeks, or developed with it
	2. headache is localized around the ipsilateral brow and eye
D.	Not better accounted for by another ICHD-3 diagnosis.

In a study of 22 cases of THS, pain and diplopia were found in 100% and 91% cases and the pain was followed by paresis. The pain was relieved in 91% within 72 hours of treatment and no patient had complete relief from paresis.^8^ These findings were consistent with our case.

MRI brain with contrast, especially the coronal view, is a crucial diagnostic study and helps to exclude other disease processes but have low specificity.^2^ Yousem et al. examined 11 patients and reported pathological MRI findings in the cavernous sinus in nine. In six of these nine the affected cavernous sinus was enlarged; in five of nine, it had a convex lateral wall. Extension into the orbital apex was seen in eight patients.^9^

High-dose glucocorticoids are the first-line treatment for Tolosa-Hunt syndrome considering its inflammatory pathology.^10^ It causes rapid resolution of the orbital pain within 1-3 days, which also serves as diagnostic confirmation. Our patient responded similarly with a significant reduction of pain in 72 hours. In one study, 40% of patients achieved pain relief within 72h and 78% within a week. In contrast, the resolution of neuropathies lags for months, which necessitates a longer course of steroids. After an initial high dose of corticosteroid, an oral taper over the course of several weeks is recommended, along with regular follow-up with subsequent MRI studies to document the resolution of the disease.^10^ Immunosuppressive drugs are the other therapeutic modality. Despite treatment, recurrences are common and the overall quality of life is poor.^1^

Tolosa-Hunt Syndrome is a rare entity with unknown etiology, presenting clinically with unilateral orbital pain and ophthalmoplegia, is a diagnosis of exclusion, which resolves spontaneously but can recur and have a dramatic response to systemic steroids. The typical clinical presentation of this case with significant response to steroids and exclusion of other condition from investigation and imaging were crucial for making the diagnosis.
